# Evaluation of almond and hazelnut reference materials for standardizing allergenic protein detection and quantification

**DOI:** 10.1007/s00216-026-06681-1

**Published:** 2026-07-24

**Authors:** Lisa E. Kilpatrick, David M. Bunk, Karen W. Phinney

**Affiliations:** 1 Biomolecular Measurement Division, National Institute of Standards and Technology, 100 Bureau Dr., Gaithersburg, MD 20899, USA

**Keywords:** Almond, Hazelnut, Allergen, Reference materials, ELISA, LC-MS/MS

## Abstract

Almonds and hazelnuts are widely consumed tree nuts; nevertheless, they are recognized by the U.S. Food and Drug Administration (FDA) and regulatory agencies in other countries as potential allergens requiring clear labeling on foods. While detecting these allergens during food production is essential, protein-based assays currently used to identify cross contact during food manufacturing often have variable results due to food processing effects, matrix interference, or antibody specificity. To address these challenges, the National Institute of Standards and Technology (NIST) Reference Materials (RMs) 8404 Almond Nut Flour for Allergen Detection and 8405 Hazelnut Flour for Allergen Detection were developed to harmonize detection methods. In this work, Dumas total protein assay and liquid chromatography-tandem mass spectrometry (LC-MS/MS) were used to characterize the protein composition of the NIST RMs along with other sources of nut flours. The RMs were next evaluated using commercial enzyme-linked immunosorbent assays (ELISAs) to assess their utility in standardizing the detection and quantification of total protein from almond and hazelnut flours. From these experiments, flours processed with different manufacturing methods were found to have similar recoveries for each ELISA only when quantifying total protein rather than total nut mass. Additionally, NIST RMs 8404 and 8405 were used to improve the reproducibility of protein recovery across commercial almond and hazelnut ELISA kits. These findings provide a framework for reporting ELISA results and using RMs to facilitate more reliable and standardized allergen risk management.

## Introduction

Approximately 3% to 4% of the population worldwide has a tree nut allergy, but that number is dependent upon the type of nut, diagnostic criteria, and geographical location [1, 2]. Increasing rates of tree nut allergies have also been reported in children [3]. Primary tree nut allergies occur when immunoglobulin E (IgE) binds to nut proteins, often causing severe reactions. On the other hand, pollen-food allergy syndrome (or oral allergy syndrome) typically results in mild symptoms and occurs when IgE, sensitized in response to pollen, cross-reacts with nut proteins. Common tree nut protein targets of IgE are seed storage proteins, profilins, oleosins, non-specific lipid transfer proteins, and pathogenesis-related (PR) proteins (including PR-5, PR-10, and PR-14) [4, 5]. Except for profilin and PR-10, the immunogenicity of the proteins tends to remain following roasting, proteolysis, or other processing [5, 6]. Although oral immunotherapy is being tested as a treatment [7], tree nut allergies are usually lifelong, requiring individuals to strictly avoid foods containing these allergens.

The FDA and other international regulatory bodies categorize tree nuts as potential allergens, requiring their explicit disclosure on food packaging. However, voluntary precautionary allergy labeling (PAL) statements may also be used by manufacturers to inform consumers that tree nuts may be present in foods. The PAL statements are unregulated in the U.S., which may lead to uncertainty about when they should be used and the safety of food products for allergic individuals. Additionally, due to the increasing prevalence of food allergies and the cost of food product recalls due to undeclared allergens [8, 9], manufacturers are under increased pressure from regulatory agencies and consumers to avoid allergen cross-contact during food production and ensure accurate labeling.

Immunoassays are commonly used to detect tree nut residues in food products [10]. While a previous study evaluated ELISA performance for detecting and quantifying almonds and hazelnuts [11], this work was done prior to the availability of reference materials to standardize results [12–14]. Recent work used a peanut butter reference material and purified proteins to evaluate commercial ELISA performance [15]; however, there remains a need to validate ELISAs specifically designed for almonds and hazelnuts. Furthermore, updated reporting guidelines for antibody-based assays now recommend measuring the mass of total allergenic protein (e.g., mg of almond protein per kg of food), rather than the total mass of the allergen [16]. This recommendation aligns with the reference doses established by the Voluntary Incidental Trace Allergen Labelling (VITAL) and Food and Agriculture Organization of the United Nations/World Health Organization (FAO/WHO) expert panels [17, 18]. Because previous studies have not addressed the new recommendations or use of reference materials, further research is needed to validate almond and hazelnut ELISA performance with these factors under consideration.

Since the Infant Formula Act of 1980 and the Nutrition Labeling and Education Act of 1990 were passed, NIST has developed a wide variety of matrix-matched Standard Reference Materials (SRMs) for foods with concentrations of different nutrients provided [19]. NIST has recently expanded its focus to food allergens. RM 8404 Almond Nut Flour for Allergen Detection [13] and RM 8405 Hazelnut Flour for Allergen Detection [14] were each produced from a single batch, then tested to ensure nutrient homogeneity and stability, and verified to be free of other nut allergens. Because all sources of bias have not been fully investigated, these RMs provide non-certified mass fractions for total protein, fat, solids, ash, and calories (carbohydrates were also provided for RM 8405) [20, 21]. RMs 8404 and 8405 are intended to be used for method development, quality control, and method harmonization.

While the amount of total protein present in the RMs was determined at the time of release, the allergenic proteins had not been extensively characterized. In this work, Dumas total protein analysis and liquid chromatography-tandem mass spectrometry (LC-MS/MS) were used to compare the protein concentrations and allergenic protein profiles of RM 8404 and RM 8405 with commercially available almond and hazelnut flours. Additionally, NIST RMs 8404 and 8405 were evaluated for their suitability in standardizing and harmonizing the performance of commercially available ELISAs.

## Methods

### Materials

RM 8404 Almond Flour for Allergen Detection and RM 8405 Hazelnut Flour for Allergen Detection were from NIST (Gaithersburg, MD). Unroasted, blanched almond flours were from commercial sources (and are referred to by the corresponding number) as follows: (1) Bob’s Red Mill (Milwaukie, OR), (2) King Arthur (Norwich, VT); (3) Mandelin (Luis Obispo, CA); (4) Pillsbury (Minneapolis, MN). Hazelnut flours with differences in processing were from commercial sources (and referred to by the corresponding number) as follows: (1) American Hazelnut Company (Gays Mills, WI); (2) Bob’s Red Mill (Milwaukie, OR); (3) Corylus Farms (Lebanon, OR); (4) Mandelin (Luis Obispo, CA). The following ELISA kits for almonds and hazelnuts were used: MonoTrace (BioFront Technologies, Tallahassee, FL), Almond or Hazelnut Residue Detection (ELISA Systems, distributed by Weber Scientific Inc., Hamilton, NJ), Alertox ELISA (Hygiena, New Castle, DE), Protein ELISA (Neogen, Lansing, MI), RIDASCREEN FAST (R-Biopharm Inc., Washington, MO), and AgraQuant Plus Allergen (Romer Labs, Union, MO). Trypsin was from Promega (Madison, WI). The C18 spin columns, trifluoroacetic acid, and LC-MS grade 0.1% formic acid in water or acetonitrile were purchased from Fisher Scientific (Pittsburgh, PA). Trizma buffer, tris(2-carboxyethyl)phosphine hydrochloride (TCEP), 2,2,2-trifluoroethanol (TFE), and iodoacetamide were purchased from Millipore Sigma (St. Louis, MO).

### Dumas total protein analysis

The commercial nut flours and unopened bags of RM 8404 and RM 8405 were sent to Eurofins Food Chemistry Testing (U.S. Madison, WI) for Dumas analysis. Each sample was analyzed in triplicate following the Association of Official Analytical Chemists (AOAC) Official Method 968.06 to determine the total nitrogen content. The total protein concentrations were calculated by multiplying total nitrogen concentrations of each sample by the Jones factor for almonds (5.18) [13] or hazelnuts (5.30) [14].

### Trypsin digestion

Nut flour samples (3 mg each) were partially defatted with hexane. After adding hexane (900 μL), samples were mixed by vortexing, centrifuged (8000 × g, 2 min), and the supernatant was removed. This step was performed three times. The solid material was then dried in a centrifugal vacuum (Centrivap, Labconco, Kansas City, MO) for 15 min and 50 mmol/L Trizma buffer (pH 8.3) was added. Samples were heated at 60 °C for 30 min following the addition of TCEP and TFE for concentrations of 15 mmol/L and 33% (v/v) in the sample, respectively. Cysteine alkylation was performed with iodoacetamide (30 mmol/L), in the dark, for 30 min. Trypsin was added for overnight digestion at 37 °C with shaking at 600 rpm (Benchmark Scientific, Sayreville, NJ). Digestion was stopped by adding trifluoroacetic acid at a final concentration of 0.5% by volume.

Samples were centrifuged (8000 × g, 10 min) and desalted using Pierce C18 spin columns following the manufacturer’s protocol. Peptides were eluted from the column with 0.1% formic acid/60% acetonitrile/39.1% water (v/v/v). Samples were dried in a centrifugal vacuum. Peptides were solubilized in 0.1% formic acid/99.9% water (v/v) for analysis by LC-MS/MS.

### LC-MS/MS analysis

The tryptic digests were injected onto a 2.1 mm × 150 mm, 3 μm, Discovery Bio Wide Pore C18 column (Sigma Aldrich, St. Louis, MO) heated to 40 °C. The LC effluent (200 μL/min) was directed to an Orbitrap Elite mass spectrometer (Thermo Fisher Scientific, Waltham, MA). The LC mobile phases were 0.1% (v/v) formic acid in water (A) and 0.1% (v/v) formic acid in acetonitrile (B). The LC gradient was increased from 1% B to 50% B over 80 min, 50% B to 95% B over 5 min, returned to 1% B over 2 min and held at 1% B for 20 min. The workflow included injecting each sample five times and collecting Fourier transform (FT) MS1 scans followed by ten MS/MS scans in the ion trap (CID or HCD). Four blank runs were performed between samples from different types of flour. No internal standard was used.

Proteome Discoverer 2.3 (Thermo Fisher Scientific) was used to analyze the data. A database was created for the analysis, which contained all currently known allergenic proteins for almonds or hazelnuts and proteins with similar amino acid sequences identified during preliminary work. The protein sequences were from the WHO/IUIS Allergen Nomenclature Database (www.allergen.org) [22] or UniProt Knowledgebase (www.uniprot.org). Trypsin and the reverse sequence database were also included in the database. Spectra were searched with a 10 ppm precursor mass tolerance, 0.6 Da product mass tolerance, and 1% false discovery rate. Only peptides with fully tryptic cleavages and up to 2 missed cleavages were considered. Fixed alkylation of cysteine and variable modifications for methionine oxidation or protein N-acetylation were included.

### ELISAs

Almond and hazelnut flour samples were prepared at approximately 500 μg/g, based on the total nut mass, in the extraction buffer from each kit. The RMs were also ground for ≈10 min in a food processor and prepared along with the other samples. The ELISA procedure was followed for each kit using an 8-channel pipet to dispense solutions. Further dilutions of the samples were prepared volumetrically with the extraction buffer so that the concentration was within the reported ELISA quantification range (see Supplementary Tables S1 and S2). The 96-well plates were measured at 450 nm using a BioTek Synergy HT (Agilent Technologies, Inc., Santa Clara, CA) plate reader with Gen5 software. Two samples of each nut flour were prepared and analyzed in duplicate on different days for each ELISA. In a separate series of experiments, the ELISA calibration standards (the most concentrated from each kit) were diluted to the same protein concentration using the extraction buffer from the ELISA used for analysis. Calibration standards were prepared twice, on different days, and analyzed in duplicate using two randomly selected ELISA kits.

The plate reader software was used to calculate sample concentrations, according to the instructions included with the ELISA kit. Data were also analyzed to ensure that the quality control requirements of the assay, if provided, were met. The percent recovery for each sample was calculated from the ratio of the protein concentration determined by the ELISA to the concentration calculated using the weighed nut mass. The percent recovery for the protein, if not determined directly from the ELISA, was calculated from the percentage of protein for both the samples and standards included with the kit (if unavailable, the mean value from the commercially available nut flours determined in these experiments was used as shown in Supplementary Tables S1 and S2). Protein recoveries were also standardized by calculating the ratio of the sample recovery to the RM recovery measured on the same day. OriginPro (OriginLab Corp., Northampton, MA) was used to perform two-way ANOVA (significance level p < 5%) and Tukey’s test. Data were anonymized and a letter was randomly assigned to each ELISA kit.

## Results and discussion

### Nut flour protein fractions

Almond and hazelnut flours were purchased from four commercial sources. Total protein fractions (the weight of total protein per weight of nut flour) of the commercial flours and RMs were determined using Dumas analysis with three replicate measurements per flour. The total protein content of RM 8404 was determined to be 23.4% (± 0.2%), a value consistent with the previously established non-certified value for this material (23.29% ± 0.46%) [13]. Across all almond flours, protein fractions ranged from 21.3% to 23.4%, with an overall mean of 22.5% (Table 1, see the “Materials” section for manufacturer details). Measurements for each individual almond flour were repeatable, with CVs ≤ 0.9%. The low coefficient of variation (CV) of 4.8% between sources suggests uniform total protein content [23], likely attributable to similar processing (RM 8404 and the other flours were made from blanched, unroasted almonds).

The total protein fractions of the hazelnut flours from commercial sources along with RM 8405 were determined by Dumas analysis (Table 2, see the “Materials” section for manufacturer details). These flours were ground from the whole nut, except for source 3, which had partial removal of the skin. Other differences in processing are summarized in Table 2 and included roasting (sources 1 and 3, RM 8405) or partial fat removal (source 1 and RM 8405). The protein fraction measured for RM 8405, 33.8% (± 0.4%), was within the non-certified value (33.77% ± 0.31%) determined previously for this material [14]. The protein fractions determined for the hazelnut flours were found to be repeatable with CVs ≤ 1.2% (Table 2). However, the protein fractions for these samples had a wider range of values (11.5 to 33.8%) when compared to the almond flours. The overall CV for the hazelnut flours was 43.2%, indicating a significant difference between the sources of flours tested in these experiments. Because the two samples with the highest protein content were partially defatted, the fat content for all flours was calculated from the nutritional information provided by the manufacturers (see Supplementary Table S3). The fat content, between 11.6% and 60%, was inversely proportional to the protein fraction determined for each source. Consequently, higher protein concentrations were largely attributable to the reduction in total mass following fat removal. Other factors such as roasting, the removal of skin, or the nut variety/cultivar may have also contributed to differences in the total protein fraction measured.

### Protein allergen profiles

Protein allergens in the nut flours were identified by LC-MS/MS analysis of the samples following tryptic digestion. The digestion method was optimized on the RMs, and TFE was selected as the denaturant in this work, as it yielded allergenic protein identifications equivalent to the others tested (urea and RapiGest) while maintaining compatibility with LC-MS/MS (data not shown). Identified proteins were classified according to the standardized nomenclature from the World Health Organization and International Union of Immunological Societies (WHO/IUIS) Allergen Nomenclature Sub-Committee [22]. For almond and hazelnut flours, unique peptides confirmed the presence of multiple proteoforms, i.e., proteins with different amino acid sequences (Supplementary Tables S4 and S5, respectively). Although their individual allergenicity is uncharacterized, proteoforms with high sequence identity to the WHO/IUIS allergenic proteins were included to provide a more comprehensive comparison of the different flours.

**Fig. 1:**
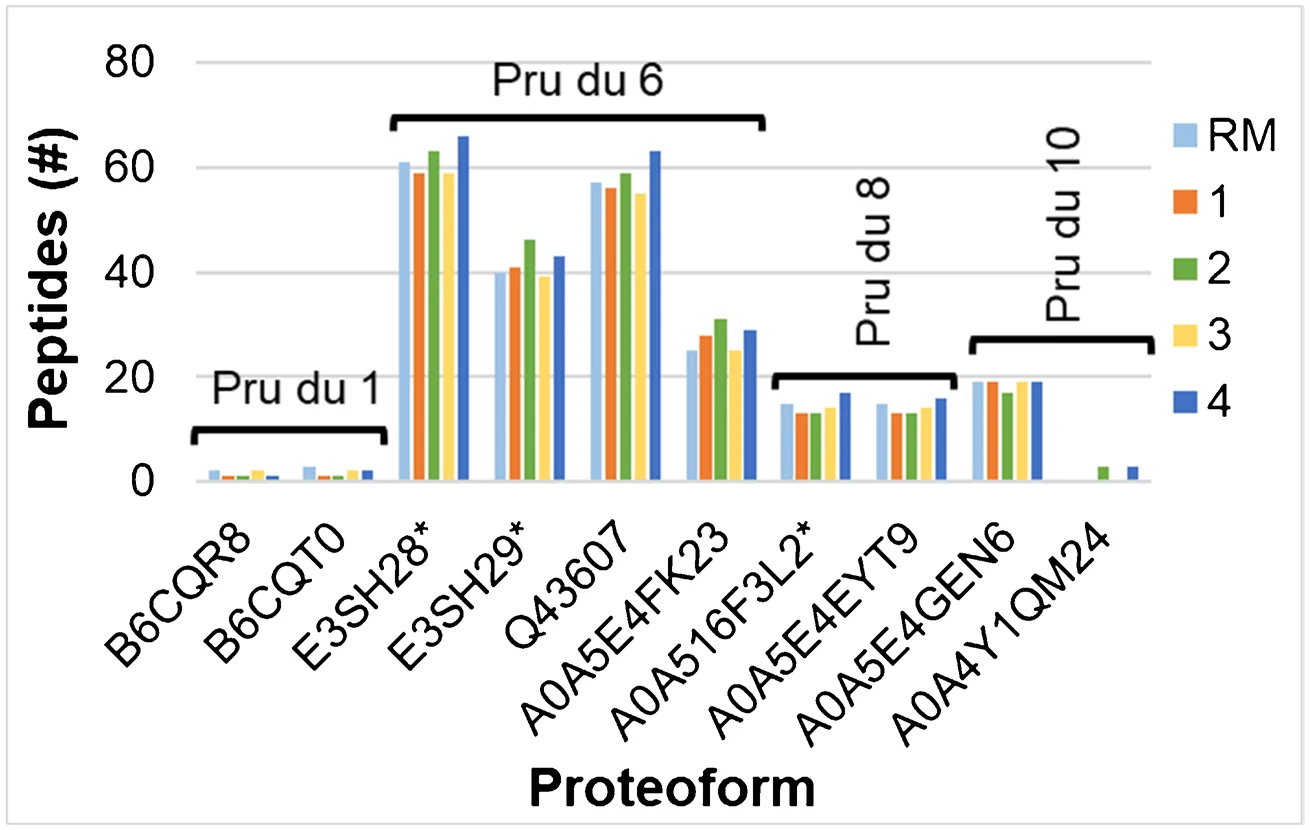
The number of total peptides (unique and shared) identified for each almond proteoform from LC-MS/MS analyses of RM 8404 and four commercial almond flours. Proteoforms are labeled using the database identifier. An asterisk (*) indicates that the protein sequence is identified as allergenic by WHO/IUIS

For the almond flours, Pru du 6 (amandin or 11S globulin) comprises ≈ 65% of the soluble almond protein [24] and was found to have the greatest number of total peptides (Fig. 1, see the “Materials” for manufacturer details). Each proteoform of Pru du 6 (E3SH28, E3SH29, Q43607, and A0A5E4FK23) had a similar number of total peptides (unique and shared) identified between RM 8404 and the other sources of almond flour. The total peptide spectrum matches (PSMs) for each protein, without corrections for protein length, were also used to estimate relative protein concentrations [25]. The semi-quantitative data for Pru du 6 also indicated a high relative concentration and showed similarities between the different almond flours for each allergenic protein (Supplementary Fig. S1).

Similar peptide numbers and PSMs were also found between the RM and commercial almond flours for the other allergenic protein groups identified as follows: Pru du 1 (PR-10), Pru du 8 (cysteine-rich antimicrobial protein), and Pru du 10 (mandelonitrile lyase 2). The proteoform of Pru du 10, A0A4Y1QM24, was only found in two samples while Pru du 3, Pru du 4, and Pru du 5 were not identified in any. These proteins may not have been identified due to having a low concentration, reduced tryptic digestion efficiency (resulting from the denaturing conditions used or the protein size/structure), or because of the genetic diversity in the almond flours analyzed.

**Fig. 2:**
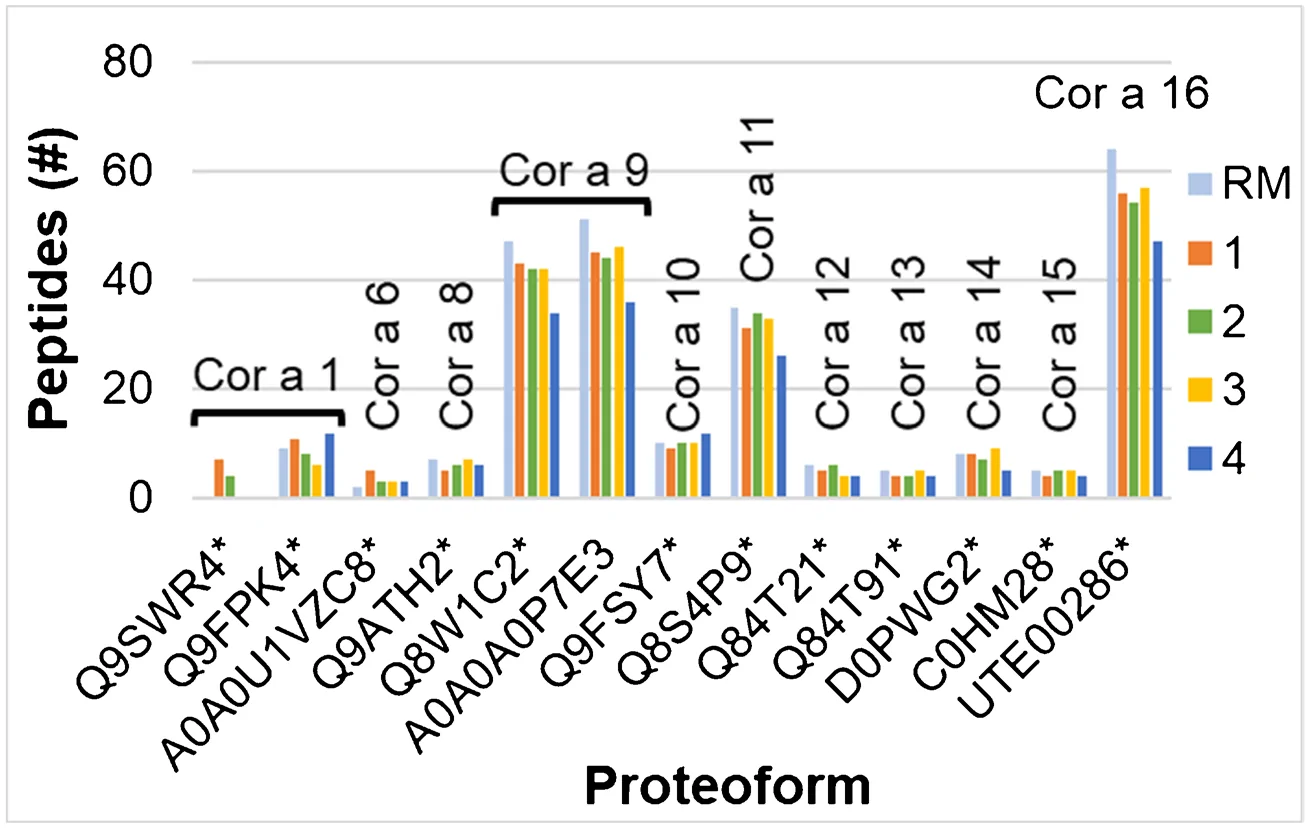
The total number of peptides (unique and shared) identified for each hazelnut proteoform from LC-MS/MS analyses of RM 8405 and four commercial hazelnut flours. Proteoforms are labeled using the database identifier. An asterisk (*) indicates that the protein sequence is identified as allergenic by WHO/IUIS

The total number of unique and shared peptides (Fig. 2) and PSMs (Supplementary Fig. S2) for RM 8405 and the different sources of hazelnut flour were then compared (see the “Materials” for manufacturer details). The related proteoforms of Cor a 9 (Q8W1C2 and A0A0A0P7E3, 11S globulin) and Cor a 11 (7S globulin) indicate high abundance in all samples which agrees with other published data [26, 27]. While the amount of Cor a 16 present in hazelnuts has not yet been reported, its N-terminus is proteolytically cleaved forming several fragments in its mature form; therefore, the number of peptides or PSMs identified does not directly indicate the concentration of this protein [28]. Also, the relatively large number of peptides found may be a result of the large size of the precursor protein for Cor a 16 (approximately 138 kDa).

Aside from Cor a 2 (profilin), all other allergenic hazelnut proteins listed on the WHO/IUIS Database were identified in RM 8405 and the hazelnut flours (Fig. 2, Supplementary Table S5) as follows: Cor a 1 (PR-10), Cor a 6 (isoflavone reductase homologue), Cor a 8 (non-specific lipid transfer protein), Cor a 10 (luminal binding protein), Cor a 12 (oleosin), Cor a 13 (oleosin), Cor a 14 (2S albumin), and Cor a 15 (oleosin). Cor a 1 (Q9SWR4) and Cor a 2 were also not consistently identified in all flours, similar to the reasons given for the almond proteins. For the flours with the lowest protein concentrations (samples 2 and 4), some proteins appear with lower numbers of peptides or PSMs; however, the numbers of peptides or PSMs do not appear to directly correlate with the concentration. Other factors may have affected the semi-quantitative data such as protein/peptide characteristics (length, number of tryptic digestion sites, LC-MS/MS ionization), loss during sample preparation, and the protein sequences in the databases used to analyze the MS/MS data (i.e., cultivars may not have been fully represented).

### ELISA recoveries for total nut

**Fig. 3:**
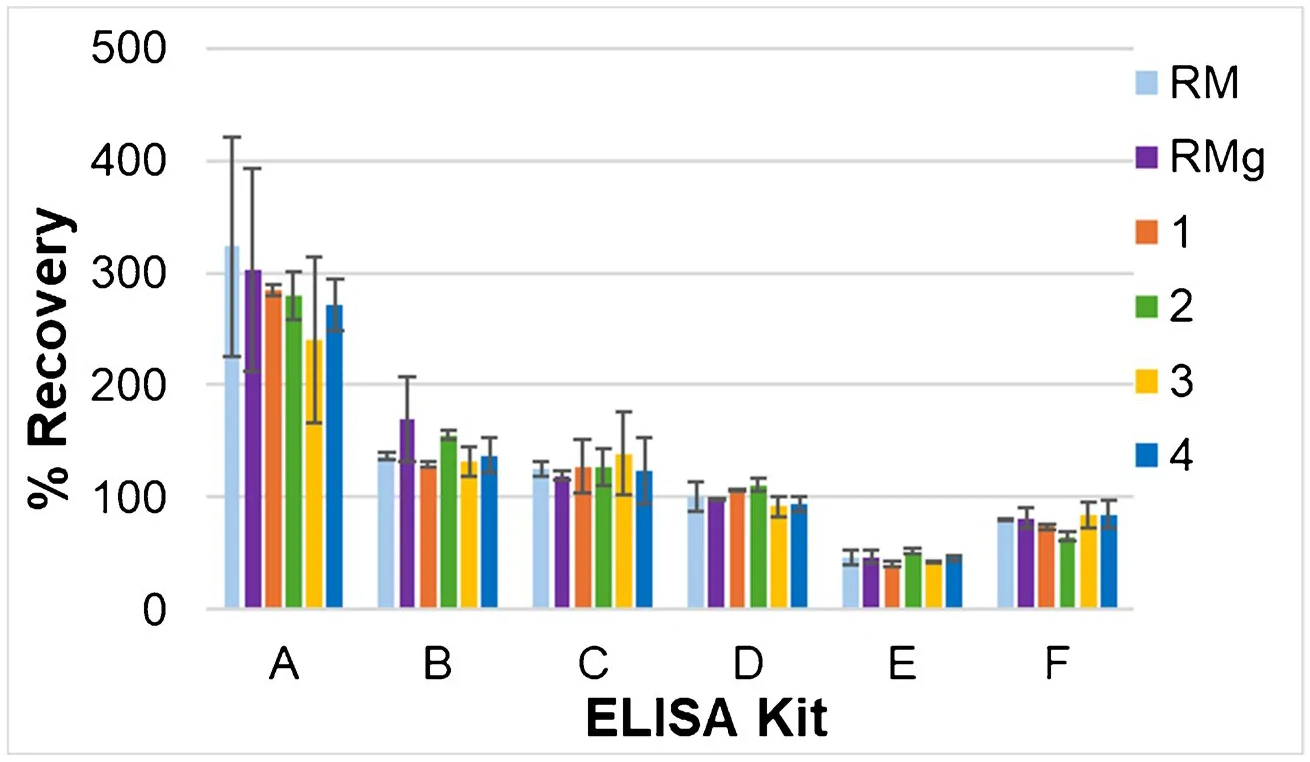
Total nut recovery of almond ELISA kits for RM 8404 and commercial almond flours. Data for RM 8404 that was further ground (RMg) is also shown. Error bars show the ± standard deviation (SD)

**Fig. 4:**
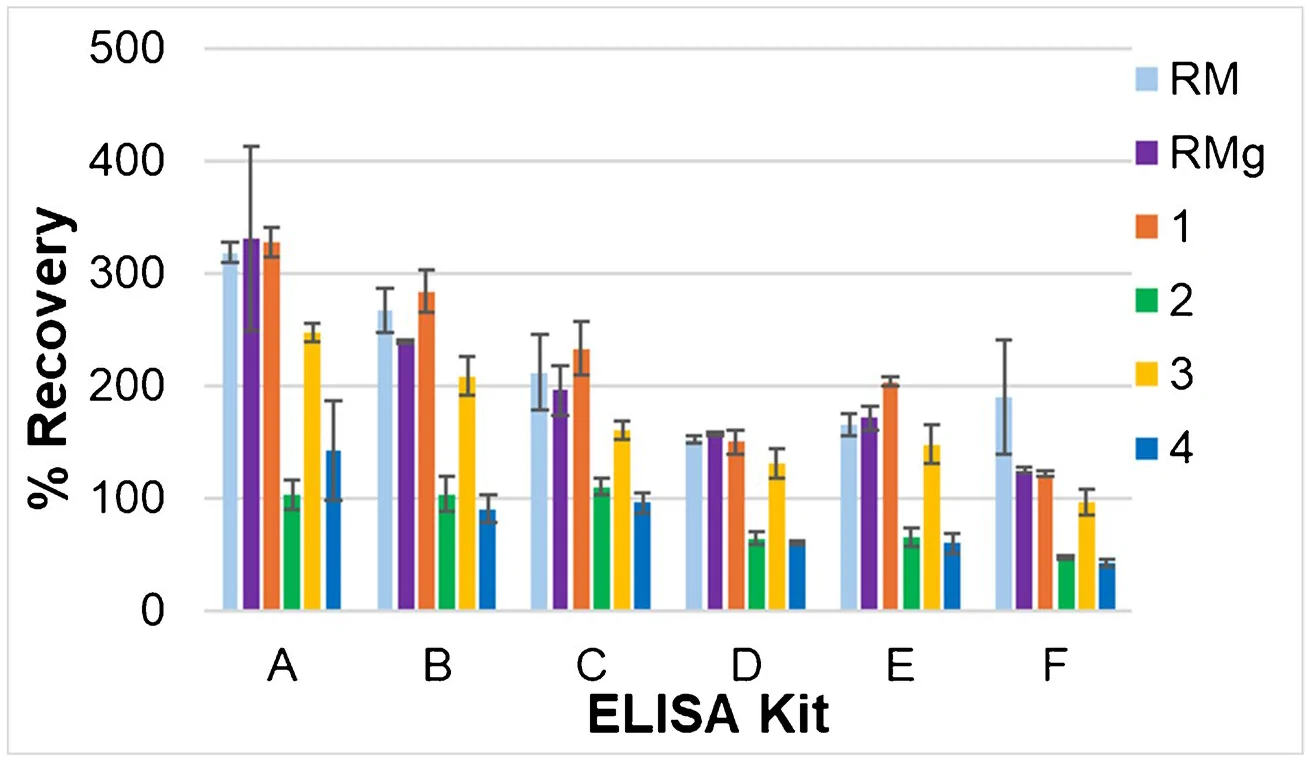
Total nut recovery of hazelnut ELISA kits for RM 8405 and commercial hazelnut flours. Data for RM 8405 that was further ground (RMg) is also shown. Error bars show ± SD

RMs 8404 and 8405 were evaluated, in comparison with almond or hazelnut flours from commercial sources, using ELISA kits from various manufacturers. The recovery of the total nut mass from the ELISA kits ranged from 41 to 323% for RM 8404 and the commercial almond flours (Fig. 3). For RM 8405 and the commercial hazelnut flours, a range of 42% to 330% was found (Fig. 4). Only a few ELISA kits performed within the 60% to 120% recovery range recommended by AOAC guidelines [29] as follows: almond ELISA kits D and F (for the RM and all flours), hazelnut ELISA kits A to E (hazelnut flours 2 and 4). While grinding food items containing nuts may be expected to improve recovery of the allergenic proteins, further grinding of the RM nut flours themselves did not significantly impact results for most ELISAs (Figs. 3 and 4, exceptions were hazelnut ELISAs B and F). Two-way ANOVA for total almond mass recovery shows there is no significant difference between the RM and other flours; however, a significant difference was found between the performance of the almond ELISA kits (p < 0.0001, F = 203.7). For total hazelnut recovery, a significant difference was found between the flours, including the RM, (p < 0.0001, F = 35.7). A significant difference was also found between the hazelnut ELISA kits (p < 0.0001, F = 24.2).

### ELISA recoveries for total protein

**Fig. 5:**
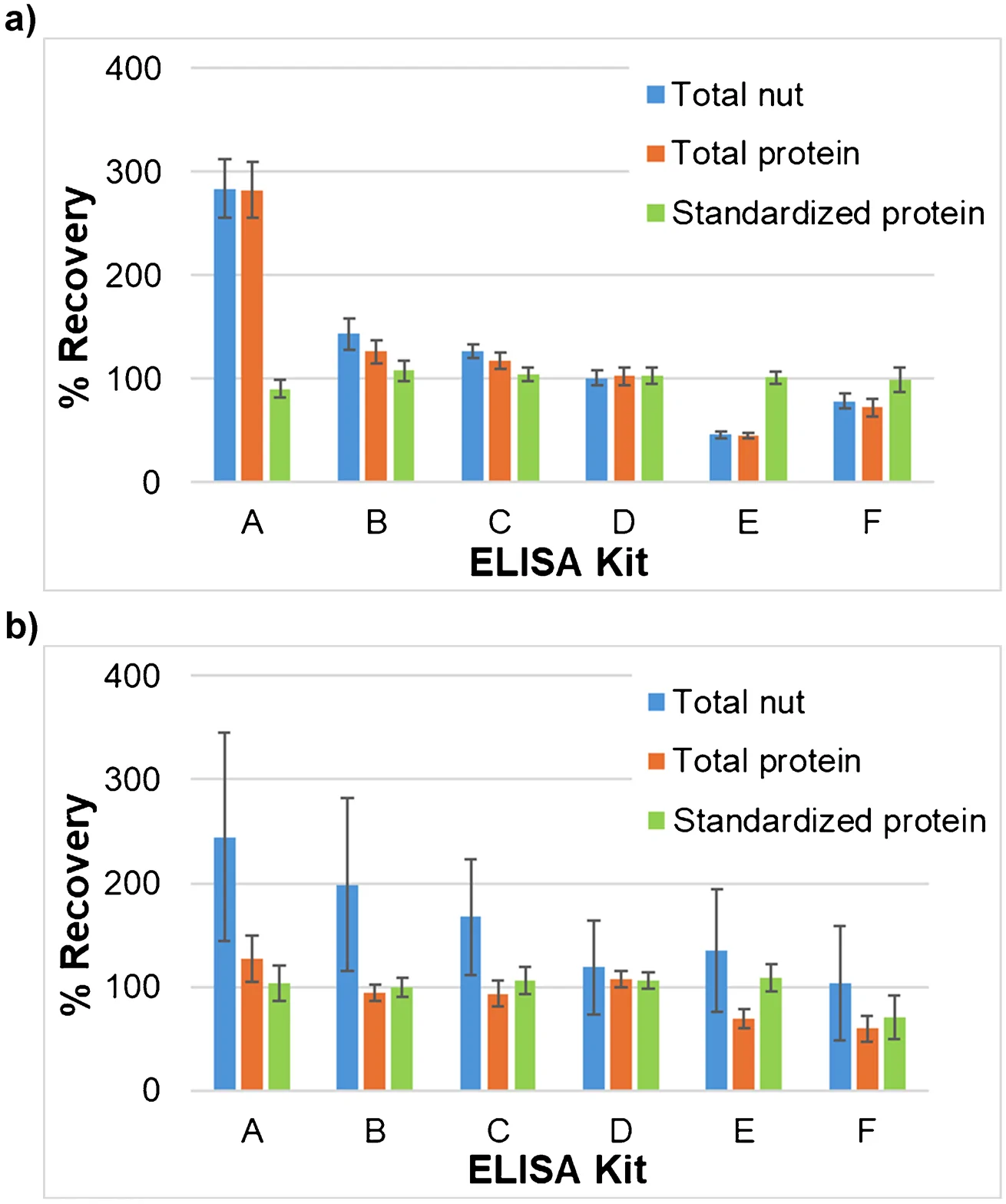
Mean ELISA recoveries for total nut, total protein and standardized total protein. Standardized protein recovery, shown as a percentage, was the ratio of ELISA value from the commercial flours to the RM. For each ELISA kit, the mean value of the RMs and flours for almonds (**a**) or hazelnuts (**b**) are shown. Error bars show ± SD

To align with clinical reference doses, recently published guidelines recommend that total protein rather than total nut mass should be reported from ELISA measurements [16]. Most ELISA kits included in these experiments followed this recommendation by providing either the protein concentrations of the calibration standards or a conversion factor to be used. For the manufacturers that did not provide this information, the protein fraction was estimated from Dumas analysis of the nut flours (i.e., 22.5% or 13% for almonds or hazelnuts, respectively, from Supplementary Tables S1 and S2). Total protein recoveries measured for each flour are shown in Supplementary Figs. S3 and S4. To more easily compare results between the ELISAs, protein recoveries of the RMs and commercial flours were calculated as a mean value for each of the kits (Fig. 5).

The mean total protein recovery of each almond ELISA was found to range from 45.6% to 282.2%, which was similar to the mean recovery based on the total almond mass (45.8% to 283.7%) shown in Fig. 5a. Two-way ANOVA showed that there was no significant difference between the mean total protein recoveries measured between the almond flours, including RM 8404, for each ELISA. These results are likely due to similar processing and protein content for the almond flours (Table 1).

For the hazelnut flours, the mean total nut recovery for each ELISA had a range of 103.6% to 244.7% (Fig. 5b). Adjusting the recoveries for the protein fraction (Table 2) reduced the range from 59.9% to 127.6% (Fig. 5b). The CVs for each hazelnut ELISA also decreased when total nut recoveries were adjusted by the protein fractions (41.8% to 13.4%, respectively). Unlike the total hazelnut recoveries, two-way ANOVA found no significant difference between the total protein recoveries of RM 8405 and the other hazelnut flours measured by each ELISA. Protein recoveries for the ELISAs also moved closer to full recovery (Fig. 5b). Therefore, the major source of variability between the hazelnut flours was due to protein content. Interestingly, no difference was detected between protein recoveries from the roasted and unroasted hazelnut flours. This may be because the antibodies are able to bind to multiple proteins present in both the unroasted and roasted flours or that they preferentially bind to a heat-stable region [6].

### Inter-assay comparisons

When the mean protein recoveries between the almond or hazelnut ELISAs were analyzed by two-way ANOVA, significant differences were found between the kits from different manufacturers (p < 0.0001, F = 231.4 and p < 0.0001, F = 26.6, respectively). Subsequently, the mean ratio of the protein recovery for the commercial flours to the RM value, measured at the same time, was calculated for each ELISA. Standardized protein recoveries for both the almond (Fig. 5a) and hazelnut (Fig. 5b) ELISAs moved closer to full recovery as ELISA means changed from 124.5% to 101.0% and 92.2% to 99.5%, respectively. These findings demonstrate that inter-assay variations, such as differences in protein extraction efficiency of the buffers or protein concentrations of the calibration standards, could be normalized by the RM.

However, two-way ANOVA of standardized protein recoveries between ELISA kits for the almonds (p = 0.04768, F = 2.6,) or hazelnuts (p = 1.1 × 10^−4^, F = 8.2,) were still significantly different. Mean values of the standardized protein recoveries for almond ELISA kits A and B were found to be grouped separately from each other by Tukey’s test; however, individually they were each grouped with results from the other kits. For the hazelnut ELISA kits, only kit F yielded a standardized protein recovery significantly different from all other kits. The kits found to be significantly different were not ones in which the protein fraction of the ELISA calibration standards were estimated from the experimental protein concentrations.

To further investigate variability between ELISA kit recoveries, one calibration standard from each manufacturer (the highest concentration) was diluted to a uniform protein concentration and analyzed using two randomly selected ELISAs. Each almond or hazelnut ELISA calibration standard tested with its own kit was found to give 100% recovery for total protein (Supplementary Fig. S5). Almond ELISA kit C also showed full protein recovery for calibration standards from kits D and F. Protein recoveries for the other calibration standards measured with the almond or hazelnut ELISAs were either above or below full recovery and appeared to be dependent upon both the calibration standard and ELISA used for the analysis.

Additional examination of the data shows that protein recoveries for the almond flours analyzed with ELISA kits A and E (Fig. 5a) appeared to be influenced by differences in protein concentration of the ELISA calibration standards that were measured. The low protein concentration in the calibration standard from kit A (Supplementary Fig. S5, mean protein recovery of 23.2%) and the high concentration in the calibration standard from kit E (Supplementary Fig. S5, mean protein recovery of 222.7%) resulted in disproportionately higher and lower recoveries reported in the almond flour samples, respectively. The differences in protein recoveries for almond flour samples analyzed with ELISA kits A and E were largely mitigated by standardizing the data to the RM value (Fig. 5a) since the RM was prepared alongside the other flour samples and similarly affected by both the protein extraction method and the calibration standards. However, even after standardization of the almond flour data, the mean protein recovery from almond ELISA kits A and B were still found to be different from each other which may be attributed to differences in antibody epitope recognition. This factor may also account for the lower protein recovery determined for hazelnut ELISA kit F (Fig. 5b). However, further conclusions cannot be made without additional studies on the specific antibodies used in these kits.

## Conclusions

The protein profiles and concentrations of RM 8404, RM 8405, and commercially available nut flours were comprehensively characterized using Dumas total protein analysis and LC-MS/MS. Similar allergenic protein profiles were found in RM 8404 and the commercial almond flours or RM 8405 and the commercial hazelnut flours analyzed in these experiments. While similar protein concentrations were found by Dumas analysis between RM 8404 and the commercial almond flours, differences were identified between the hazelnut flours. These differences were identified as a result of the processing methods used during manufacturing (primarily defatting).

When the flours were analyzed with commercial ELISAs, recoveries for the total mass or total protein of the almond flours were not significantly different between RM 8404 and the commercially available almond flours. Only upon correcting the ELISA recoveries for the percentage of protein, determined by Dumas protein analysis, were RM 8405 and the other hazelnut flours found to be similar when measured by each kit individually. Therefore, it is preferable to report the total protein mass per total food mass from the ELISA assays to improve accuracy and consistency of results for the hazelnut flours and for other samples where protein concentrations are expected to vary.

Standardizing protein recoveries of the commercial nut flours to the RM values resulted in further improvement in ELISA performance between kits. These results suggested that variation between the ELISA kits stemmed from either the protein extraction efficiency of the buffers or in the protein content of the calibration standards included in the kits. However, differences persisted for some of the almond or hazelnut ELISAs, which may be due to differences in antibody specificity and requires further investigation. These findings demonstrate that while validating ELISAs with an RM during analytical method development may be ideal, post-hoc standardization is a viable alternative for improving accuracy and reproducibility.

This study demonstrates that NIST RMs 8404 and 8405 were effective for evaluating almond and hazelnut ELISAs for the extraction, detection, and quantification of allergenic proteins and reduced variability of measurements from different manufacturers. Although RMs 8404 and 8405 are primarily intended for method development, quality assurance, and method harmonization, they can be adapted for complex food matrices. For instance, validating methods for analysis of foods where protein modification is expected to occur, such as during baking or in samples with polyphenols, may require developing an in-house reference material by spiking the RM into the specific food matrix and replicating the processing conditions [30]. While matrix-specific experiments were outside the scope of this study, it was essential to first understand the comparability of the assays within simpler matrices before extending RM applications to more diverse, complex food matrices and processing methods in future research. Additionally, though this work evaluated six commercially available ELISAs for each nut, these RMs may be used to validate and harmonize other quantification methods such as LC-MS/MS. Ultimately, integrating RMs into testing workflows will allow laboratories to harmonize analytical measurements, improving the reliability of food labeling and consumer protection.

## Supplementary Material

Supp1

**Supplementary Information** The online version contains supplementary material available at https://doi.org/10.1007/s00216-026-06681-1.

## Figures and Tables

**Table 1:** Total protein fractions (%) for RM 8404 and commercial almond flours determined by Dumas analysis. All flours were blanched prior to milling. The mean, standard deviation (SD), and percent coefficient of variation (% CV) determined from triplicate measurements of each sample are shown

Sample	Mean	SD	% CV
RM 8404	23.4	0.2	0.9
1	21.4	0.1	0.3
2	23.6	0.1	0.2
3	22.8	0.2	0.7
4	21.3	0.1	0.5
All samples	22.5	1.1	4.8

**Table 2:** Total protein fractions (%) for hazelnut flours determined by Dumas analysis. The mean, standard deviation (SD), and percent coefficient of variation (% CV) determined from triplicate measurements of each sample are shown. Differences in the flour processing are also shown

Sample	Processing	Mean	SD	% CV
RM 8405	Roasted, partially defatted	33.8	0.4	1.2
1	Roasted, partially defatted	32.0	0.2	0.5
3	Roasted, partial skin removal	24.8	0.2	0.6
2	Unroasted	14.5	0.1	0.4
4	Unroasted	11.5	0.1	0.9
All samples		23.3	10.1	43.2

## Data Availability

Data are available at the following location: https://doi.org/10.18434/mds2-4200.
