# Supplementary material for: Evaluation of almond and hazelnut reference materials for standardizing allergenic protein detection and quantification
Source: Anal Bioanal Chem. Author manuscript; Available in PMC 2026 Sep 14. (PMC13573197; doi:10.1007/s00216-026-06681-1)

Supplementary Material for:

Evaluation of almond and hazelnut reference materials for standardizing allergenic protein detection and quantification

Lisa E. Kilpatrick[1], David M. Bunk[1], Karen W. Phinney[1]

[1]National Institute of Standards and Technology, Biomolecular Measurement Division, 100 Bureau Dr., Gaithersburg, MD 20899, U.S.

Corresponding author: lisa.kilpatrick@nist.gov

Contents

Table S1 Quantification ranges for the almond ELISA kits 3

Table S2 Quantification ranges for the hazelnut ELISA kits 3

Table S3 Fat content of the hazelnut flours. 4

Table S4 Almond allergenic proteins and related proteoforms identified in RM 8404 and other flours 4

Table S5 Hazelnut allergenic proteins and related proteoforms identified in RM 8405 and other flours 5

Fig. S1 The number of PSMs identified for each almond proteoform 6

Fig. S2 The number of PSMs identified for each hazelnut proteoform............................ 6

Fig. S3 Total protein recovery of almond ELISA kits for RM 8404 and commercial almond flours 7

Fig. S4 Total protein recovery of hazelnut ELISA kits for RM 8404 and commercial hazelnut flours 7

Fig. S5 Protein recoveries of ELISA calibration standards evaluated with different ELISA kits 8

Table S1 Quantification ranges for the almond ELISA kits. The sample dilution factor (DF) from the ELISA protocol was used to calculate the quantification ranges of total nut and total protein in the well plate.

Manufacturer	Kit	Quantification Range for total nut (ppm)	DF	% Protein in standard	Total Nut Range (ppb)	Total Protein Range (ppb)
BioFront	MonoTrace	1-40	40	22.5[a]	25-1000	6-225[b]
ELISA Systems	Almond Residue	2.4-24	10	21	240-2400	50-500
Hygiena	Alertox	0.5-10	20	20.8	25-500	5-104
Neogen	Protein Rapid Kit	0.01-0.27[c]	none	22.5[a]	44-1200[b]	10-270[c]
R-Biopharm	RIDASCREEN FAST	2.5-20	20	23	125-1000	29-230
Romer Labs	AgraQuant Plus	1-25	20	20	20-1250	10-250

[a]Value not provided by manufacturer; value shown was estimated from the almond flours analyzed in these experiments (Table 1).
[b]Calculated using the experimental percent protein shown in the table.
[c]Total protein range provided by the manufacturer.

Table S2 Quantification ranges for the hazelnut ELISA kits. The sample dilution factor (DF) from the ELISA protocol was used to calculate the quantification ranges of total nut and total protein in the well plate.

Manufacturer	Kit	Quantification Range for total nut (ppm)	DF	% Protein in standard	Total Nut Range (ppb)	Total Protein Range (ppb)
BioFront	MonoTrace	1-40	40	13[a]	25-1000	3-130[b]
ELISA Systems	Hazelnut Residue	3.7-37	10	13.5	370-3700	50-500
Hygiena	Alertox	1-40	20	13.2	50-2000	7-263
Neogen	Protein Rapid Kit	0.01-0.8[c]	none	13[a]	77-6231[b]	10-810[c]
R-Biopharm	RIDASCREEN FAST	2.5-20	20	15	125-1000	19-150
Romer Labs	AgraQuant Plus	2-25	20	12	100-1250	12-150

[a]Value not provided by manufacturer; value shown was estimated from the unroasted hazelnut flours analyzed in these experiments (Table 2).
[b]Calculated using the experimental percent protein shown in the table.
[c]Total protein range provided by the manufacturer.

Table S3 Fat content of the hazelnut flours. Values were calculated from the nutritional information on the package provided by the manufacturer (see Materials for manufacturer details).

Sample	% Fat
RM 8405	11.6
1	15.0
3	42.9
2	50.0
4	60.0

Table S4 Almond allergenic proteins and related proteoforms identified in RM 8404 and other flours. The standard allergen name is shown along with the biochemical name, database identifier, % identity (calculated compared to the first proteoform listed using SIM, web.expasy.org/sim/), and number of amino acids. Proteins listed by WHO/IUIS as allergenic are indicated with an asterisk (*). Protein IDs in bold were identified by LC-MS/MS.

Allergen	Biochemical name	Database ID	% Identity (AA #)
Pru du 1	PR-10, Bet v 1-related protein	B6CQS9*<br>**B6CQT0**<br>**B6CQR8**	100 (160)<br>96.9 (160)<br>73.8 (160)
Pru du 3	Non-specific lipid transfer protein type 1 (nsLTP1)	C0L0I5*	100 (123)
Pru du 4	Profilin	Q8GSL5*<br>A0A5E4FBP1	100 (131)<br>100 (182)
Pru du 5	60s acidic ribosomal protein P2	Q8H2B9*<br>A0A5E4F275	100 (113)<br>100 (113)
Pru du 6	Amandin, 11S globulin legumin-like protein	**E3SH28***<br>A0A5E4FFS0<br>**Q43607**<br>**A0A5E4FK23**<br>**E3SH29***	100 (551)<br>99.6 (551)<br>99.1 (551)<br>61.4 (317)<br>58.8 (504)
Pru du 8	Alpha-hairpinin; Cysteine rich antimicrobial protein	**A0A516F3L2***<br>**A0A5E4EYT9**	100 (264)<br>99.6 (261)
Pru du 10	Mandelonitrile lyase 2; Hydroxynitrile lyase	Q945K2*<br>**A0A5E4GEN6**<br>**A0A4Y1QM24**	100 (563)<br>99.6 (563)<br>71.9 (553)

Table S5 Hazelnut allergenic proteins and related proteoforms identified in RM 8405 and other flours. The accepted allergen name is shown along with the biochemical name, database identifier, % identity (calculated compared to the first proteoform listed using SIM, web.expasy.org/sim/) and number of amino acids. Proteins listed by WHO/IUIS as allergenic are indicated with an asterisk (*). Protein IDs in bold were identified by LC-MS/MS.

Allergen	Biochemical Name	Database ID	% Identity (AA #)
Cor a 1	Pathogenesis-related protein, PR-10, Bet v 1 family member	**Q9SWR4***<br>Q9FPK2*<br>Q9FPK3*<br>**Q9FPK4***<br>Q39454*<br>A0AA49EHN2*<br>Q39453*<br>A0AA49EFQ6*<br>Q08407*<br>A0AA49X970*<br>A0AA49XEK8*<br>A0AA49X9K5*	100 (161)<br>98.8 (161)<br>97.5 (161)<br>96.9 (161)<br>71.2 (160)<br>70.6 (159)<br>70.0 (160)<br>68.8 (161)<br>63.8 (160)<br>59.6 (166)<br>52.5 (160)<br>50.6 (162)
Cor a 2	Profilin	Q9AXH5*<br>Q9AXH4*	100 (131)<br>98.5 (131)
Cor a 6	Isoflavone reductase homologue	**A0A0U1VZC8***	100 (308)
Cor a 8	Non-specific lipid transfer protein type 1 (nsLTP1)	**Q9ATH2***	100 (115)
Cor a 9	11S seed storage globulin (legumin-like)	**Q8W1C2***<br>**A0A0A0P7E3**	100 (515)<br>96.5 (514)
Cor a 10	Luminal binding protein	**Q9FSY7***	100 (668)
Cor a 11	7S seed storage globulin (vicilin-like)	**Q8S4P9***	100 (448)
Cor a 12	17 kDa oleosin	**Q84T21***	100 (159)
Cor a 13	14-16 kDa oleosin	**Q84T91***	100 (140)
Cor a 14	2S albumin	**D0PWG2***	100 (147)
Cor a 15	Oleosin	**C0HM28***	100 (169)
Cor a 16	7S globulin seed storage protein (vicilin)	**UTE00286***	100 (1133)

Fig. S1 The number of PSMs identified for each almond proteoform. Proteoforms are shown using the database identifier (ID). RM 8404 and different sources of almond flour were analyzed. An asterisk (*) following the protein ID indicates that the protein sequence is identified as allergenic on the WHO/IUIS website.

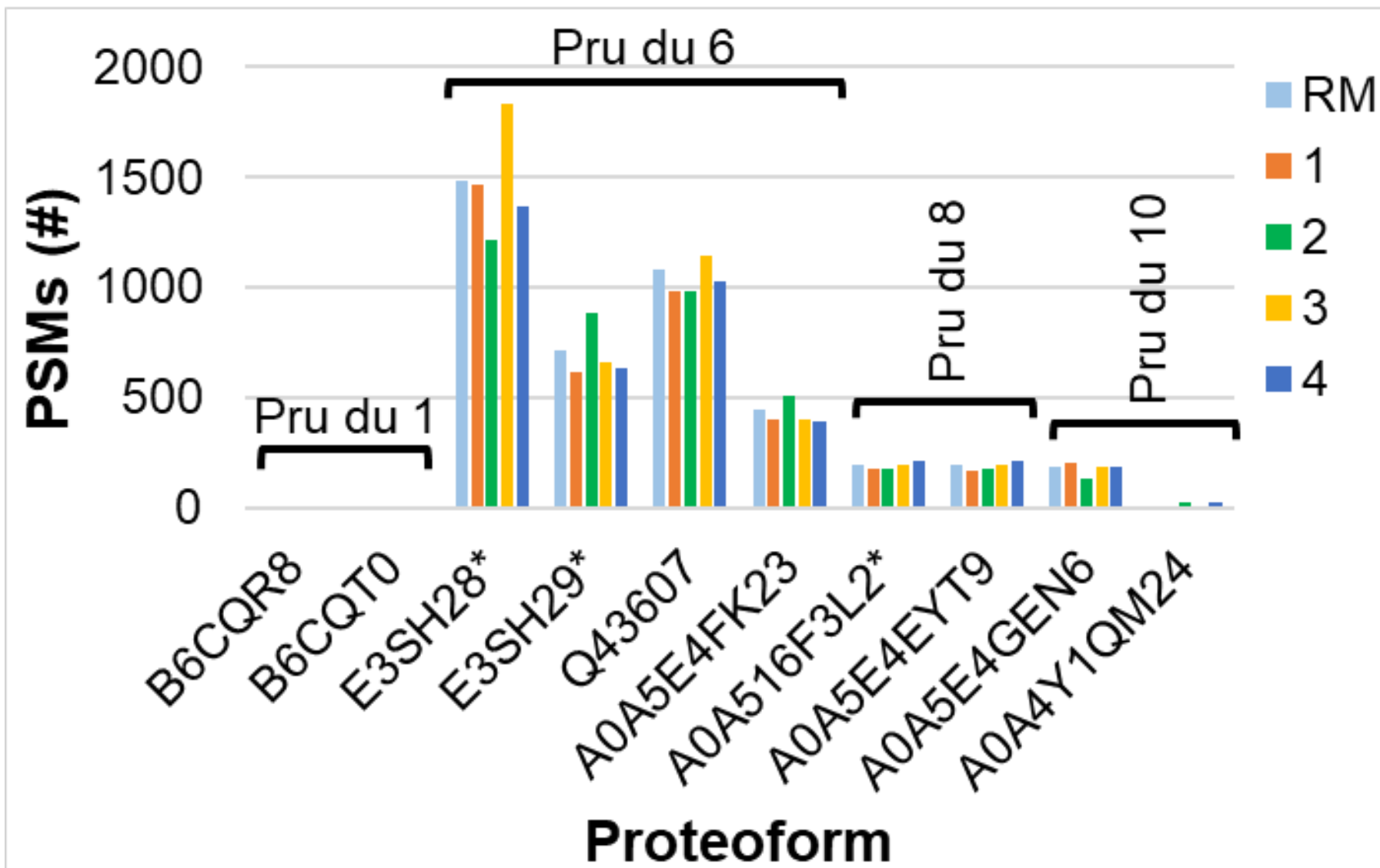

Fig. S2 The number of PSMs identified for each hazelnut proteoform. Proteoforms are shown using the database identifier (ID). RM 8405 and different sources of hazelnut flour were analyzed. An asterisk (*) following the protein ID indicates that the protein sequence is identified as allergenic on the WHO/IUIS website.

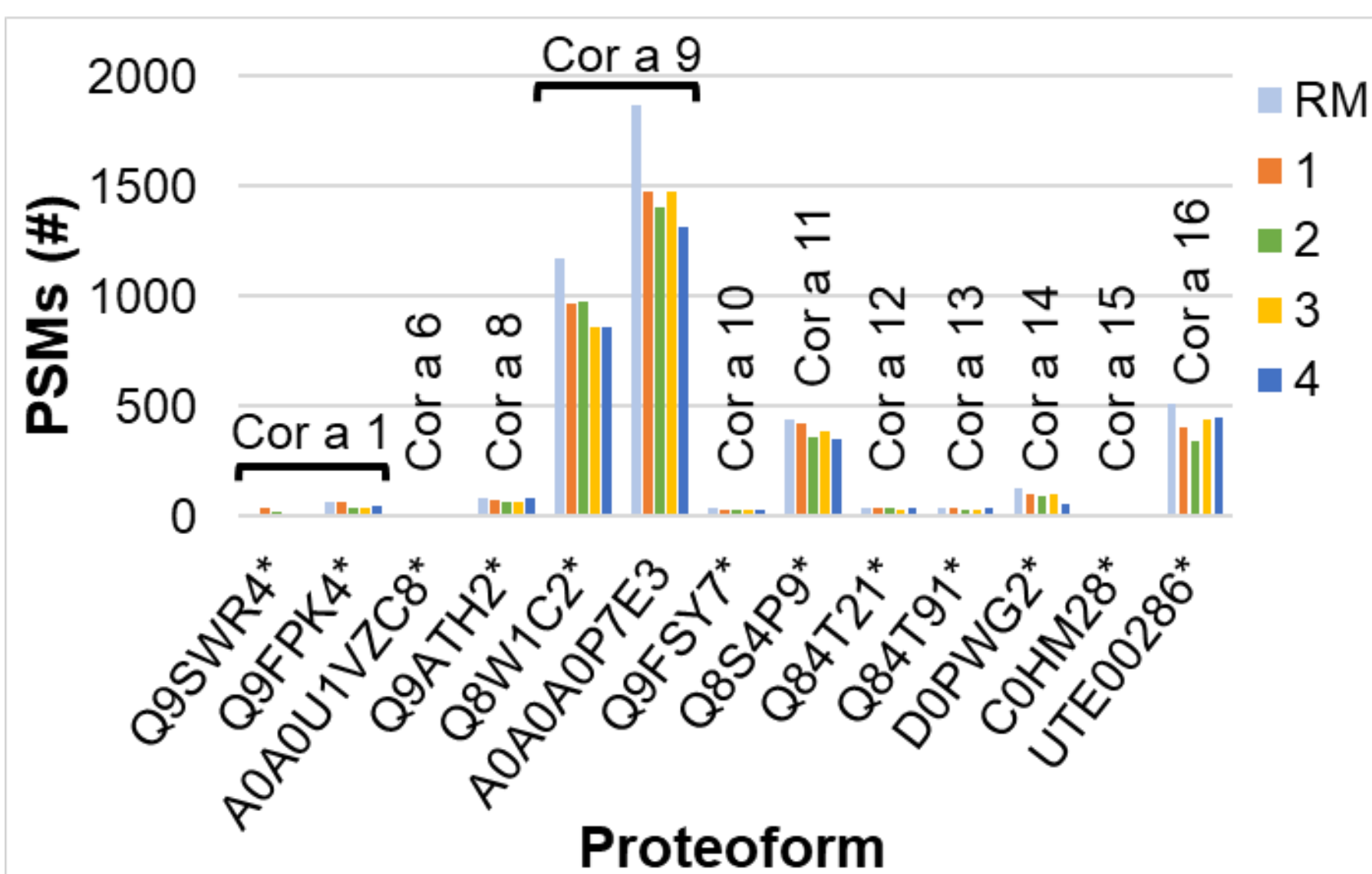

Fig. S3 Total protein recovery of almond ELISA kits for RM 8404 and commercial almond flours. Error bars show ± the standard deviation (SD).

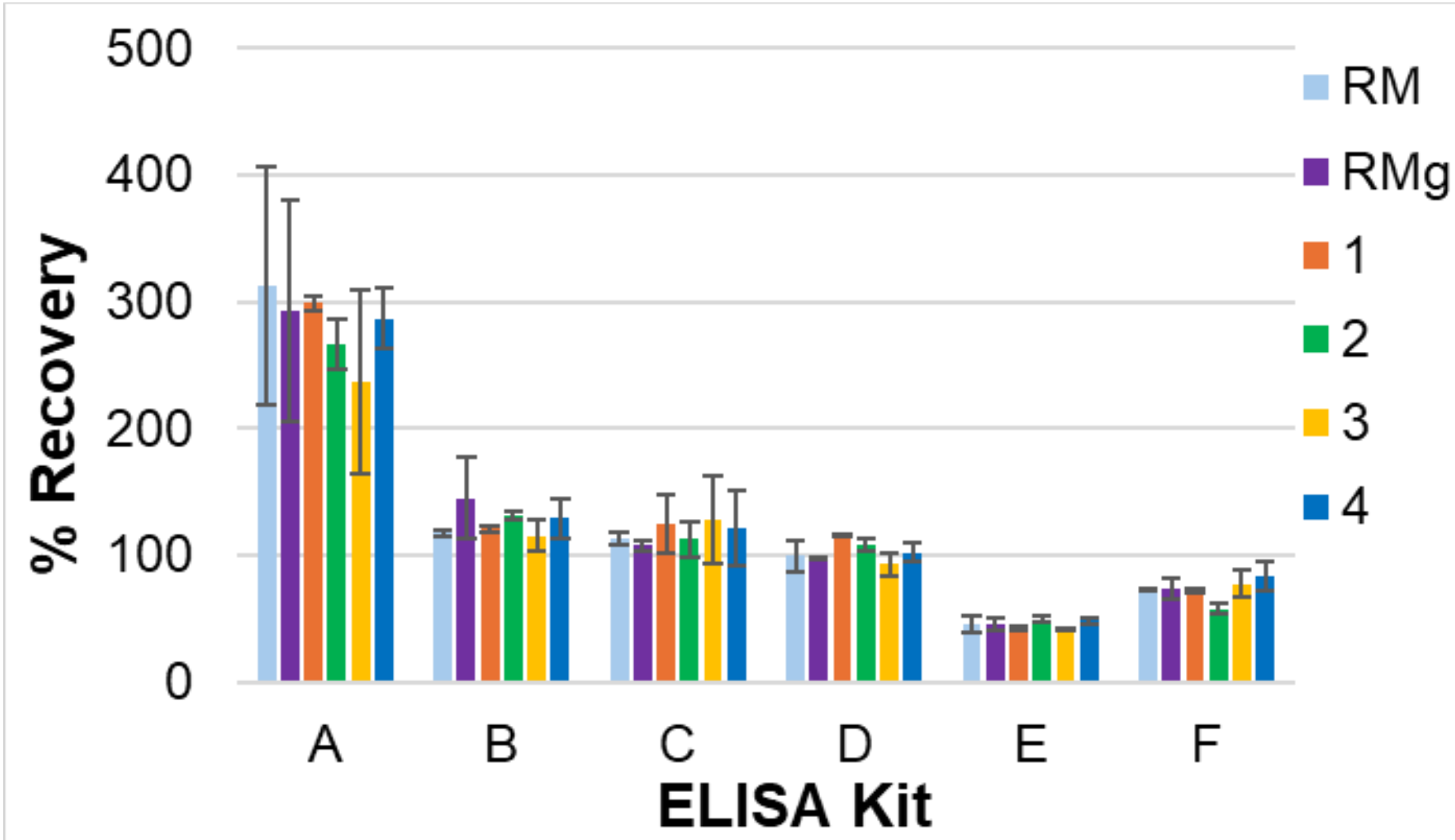

Fig. S4 Total protein recovery of hazelnut ELISA kits for RM 8404 and commercial hazelnut flours. Error bars show ± the standard deviation (SD).

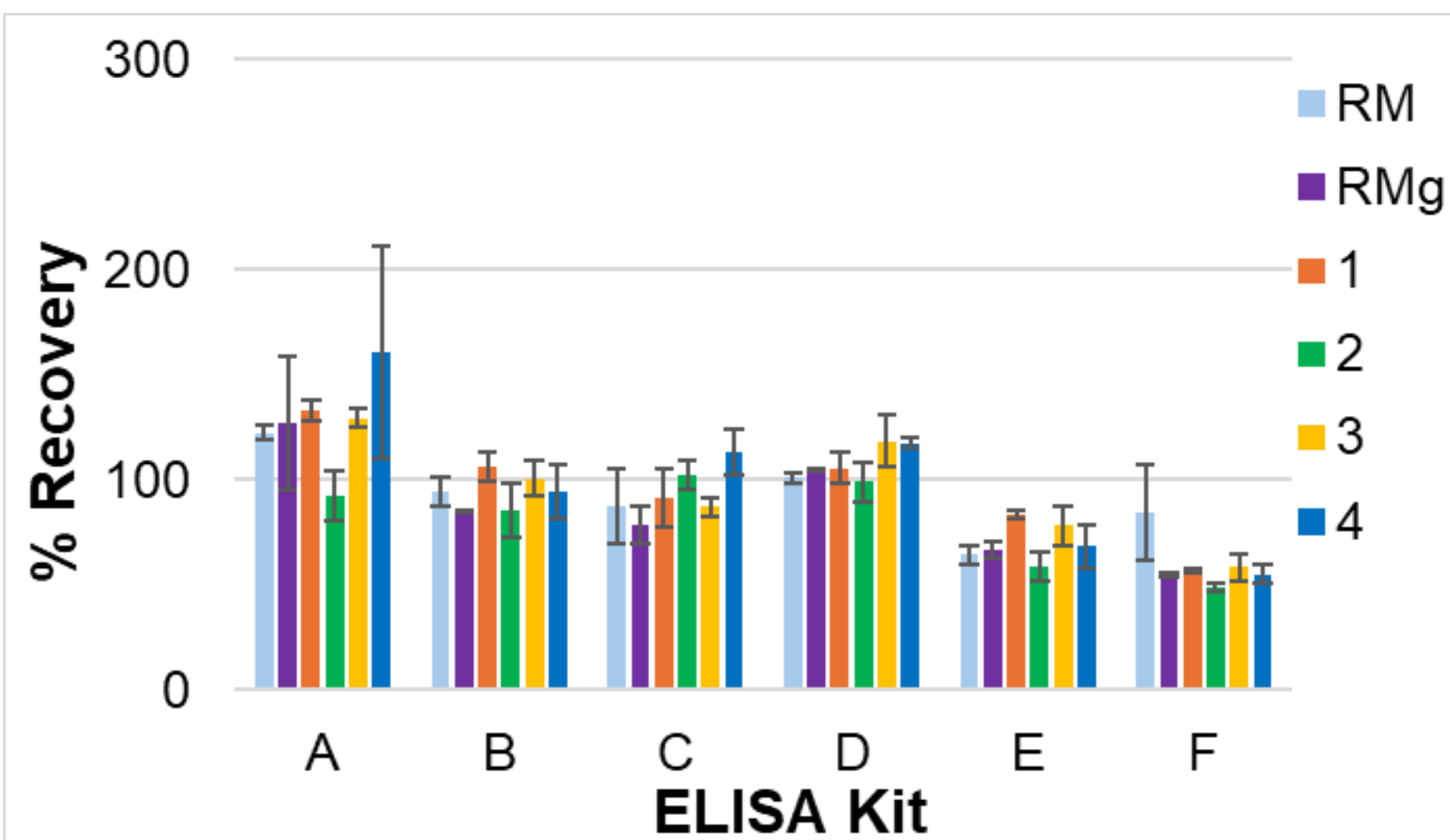

Fig. S5 Protein recoveries of ELISA calibration standards evaluated with different ELISA kits. The calibration standard with the highest concentration from each ELISA kit was selected for analysis. Calibration standards were diluted in extraction buffer to a uniform concentration and analyzed in duplicate with two different ELISA kits on two days. Results for almond (a) and hazelnut (b) calibration standards from each kit are shown. Error bars are ± SD.

a)

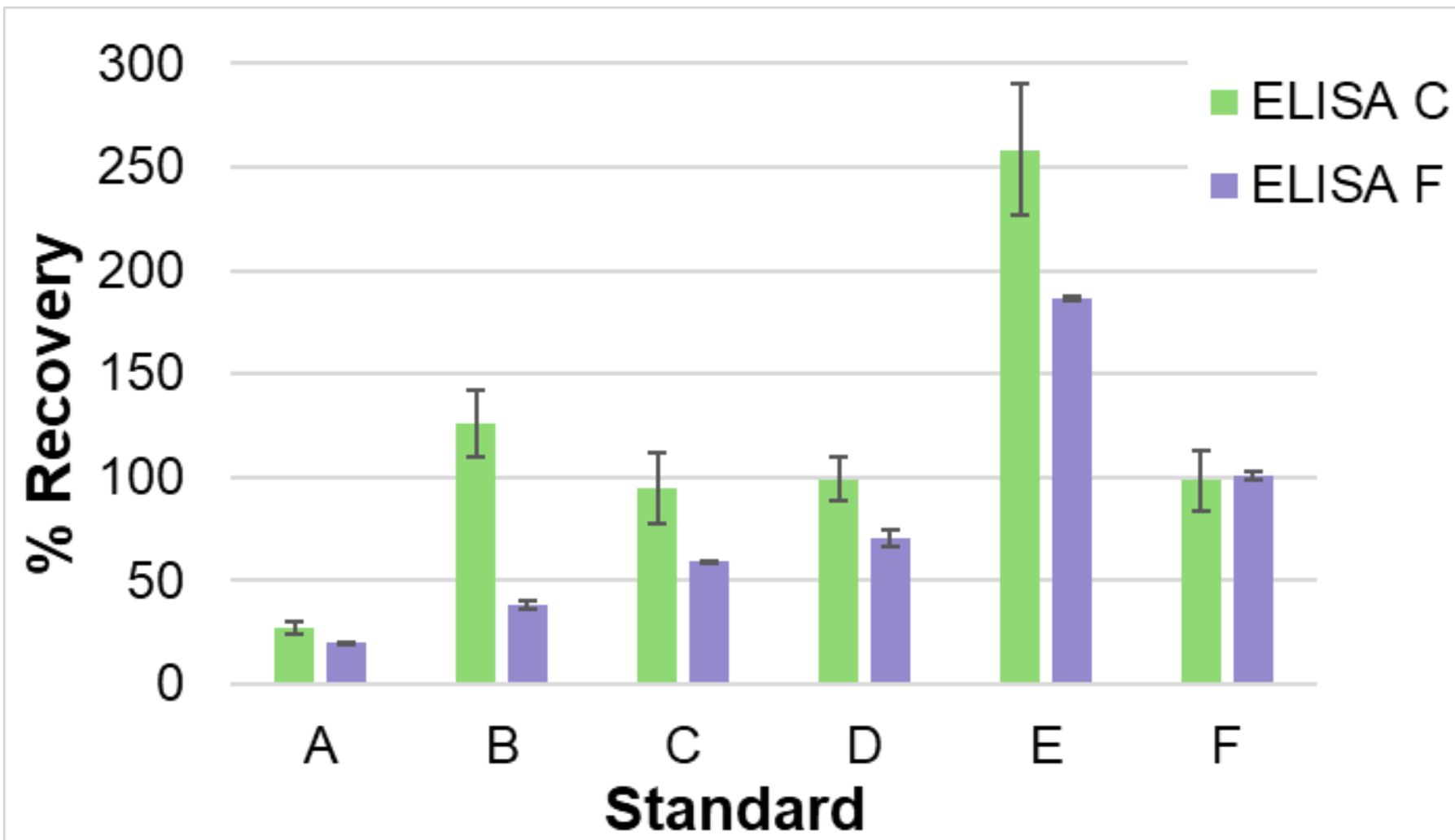

b)

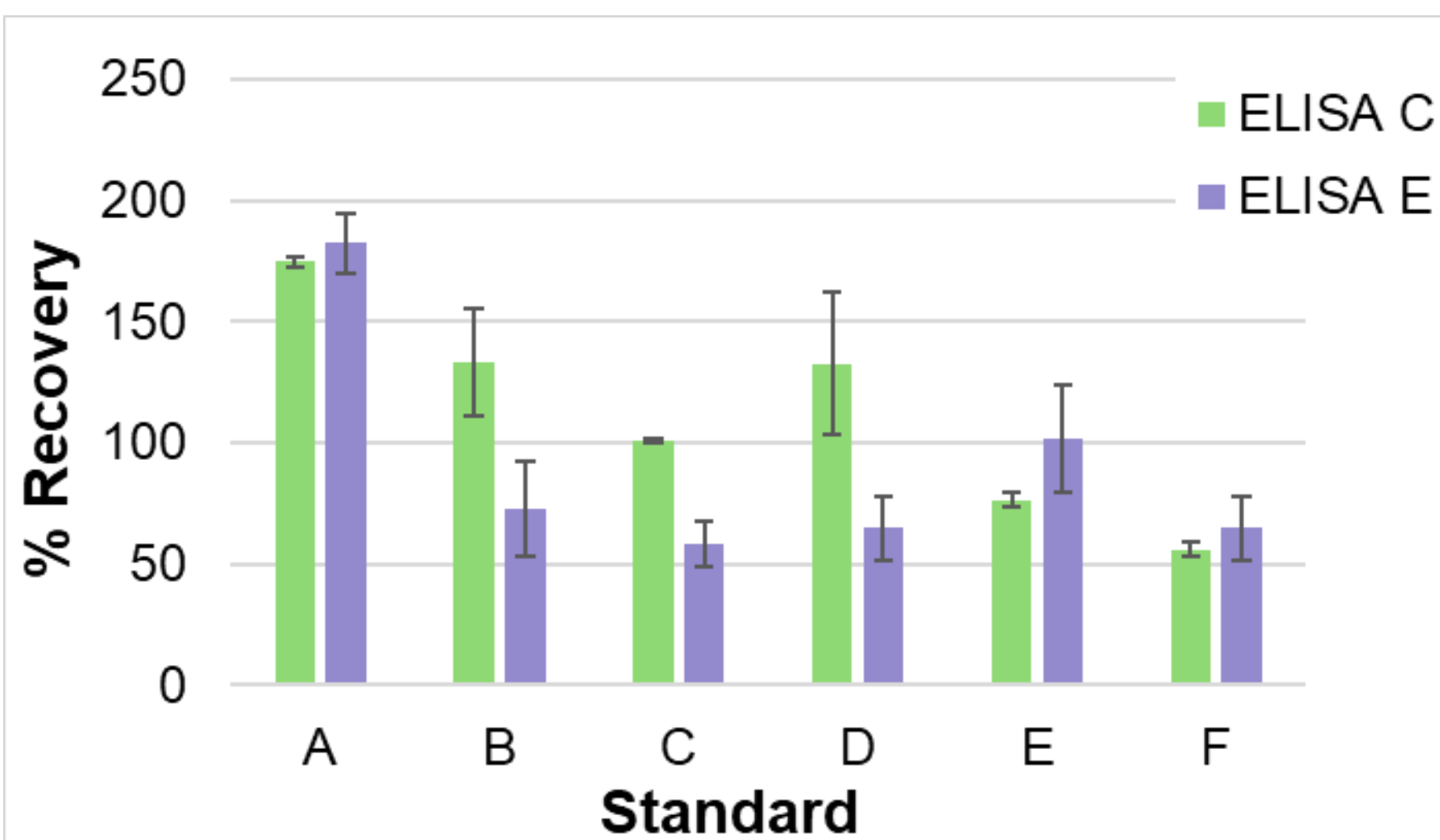

Supplement: Supplementary file 1 [file NIHMS2205448-supplement-Supp1.pdf]
